# Microbial and heavy metal contamination in herbal medicine: a prospective study in the central region of Saudi Arabia

**DOI:** 10.1186/s12906-023-04307-y

**Published:** 2024-01-02

**Authors:** Sarah F. Alharbi, Ameerah I. Althbah, Amal H. Mohammed, Mishaal A. Alrasheed, Mukhtar Ismail, Khaled S. Allemailem, Abdullah M Alnuqaydan, Ahmed Mohammed Baabdullah, Azzam Alkhalifah

**Affiliations:** 1 Department of Medical Laboratories, College of Applied Medical Sciences, Qassim University, 51452 Buraydah, Saudi Arabia; 2 Department of Laboratory and Blood Bank, King Fahd Specialist Hospital, 52211 Buraydah, Kingdom of Saudi Arabia; 3 Department of Chemistry, College of Science and Arts, Qassim University, Al-Rass, Kingdom of Saudi Arabia; 4 Department of Medical Biotechnology, College of Applied Medical Sciences, Qassim University, Buraydah, Saudi Arabia; 5 Department of Dermatology, College of Medicine, King Abdulaziz University, Jeddah, Saudi Arabia; 6 Department of Medicine, Unaizah College of Medicine and Medical Sciences, Qassim University, Unaizah, Saudi Arabia

**Keywords:** Complementary and alternative medicine, Drug safety, Infection risk, Herbal medicine, Heavy metals, Microbial contamination, Plant extract, Public health practice

## Abstract

**Introduction:**

Herbal medicine is a medical system based on the utilization of plants or plant extracts for therapy. The continual increase in global consumption and the trade of herbal medicine has raised safety concerns in many regions. These concerns are mainly linked to microbial contamination, which could spread infections with multi-resistant bacteria in the community, and heavy metal contamination that may lead to cancers or internal organs’ toxicity.

**Methods:**

This study was performed using an experimental design. A total of 47 samples, herbal medicine products sold in local stores in Qassim region, were used in the experiments. They were tested for bacterial contamination, alongside 32 samples for heavy metal analysis. Bacterial contamination was determined by the streak plate method and further processed to determine their antimicrobial susceptibility patterns using MicroScan WalkAway96 pulse; heavy metals were determined using a spectrometer instrument.

**Results:**

A total of 58 microorganisms were isolated. All samples were found to be contaminated with at least one organism except three samples. The majority of the isolated bacterial species were gram negative bacteria, such as Klebsiella spp., Pseudomonas spp. and *E. coli*., which could be of fecal origin and may lead to pneumonia, skin, or internal infections. Furthermore, most of the gram-positive bacteria were found to be multi-drug resistant. Moreover, for heavy metals, all samples had levels exceeding the regulatory limits.

**Conclusion:**

This study demonstrated the presence of bacteria and heavy metals in samples of herbal medicines. Using these contaminated products may spread resistant infections, metal toxicities, or even cancers in the community.

## Introduction

Complementary and Alternative Medicine (CAM) is defined as the use of different types of experience-based products or practices to treat or prevent diseases. It has been used for thousands of years [1] and is sometimes referred to as traditional medicine [2]. In the USA, CAM is commonly practiced by the population, with natural medicine being the most commonly used form [3]. In Saudi Arabia, herbal medicine and spiritual therapies are the most used forms of CAM [4]. In some reports, up to 94% of Saudi adults have used herbal medicine in their life [5].

As a non-conventional form of medicine, natural and herbal medicine products do not have studies to support their safety and regulate precise dosages. In addition, the collection, storage, and preparation of herbal medicine does not usually ensure strict safety and sterility regulations. As a result, herbal medicine may be exposed to microorganisms from the soil, water, inappropriate handling or storage practices and environmental factors during harvesting and storage such as rainfall or high humidity [6].

Heavy metal contamination is another serious risk of herbal medicine. Various industrial, agricultural, medical and technological applications have led to the tremendous distribution of heavy metals in the environment, including plants, soil and water. Several studies from different continents have shown that heavy metals are commonly found in herbal medicine products in concentrations exceeding the permitted limits [7]. These metals could be carcinogenic and may also lead to heart, lung, brain, and kidney toxicities [7].

In Saudi Arabia, over 80% of the studied population reported that they have used traditional recipes of herbal medicine [8]. These recipes could be in the form of orally taken products or topical creams/liquids. The use of CAM has been reported to be common among patients with difficult to treat conditions such as diabetes, cancers and allergies [9–11]. Herbal medicine products are usually made with non-sterile methods. Studies from different countries have already shown the microbial contamination of these products [12, 13]. Being commonly used in Saudi Arabia, this would lead to spread of serious infections and may carry huge risks on the public health if these are shown to be contaminated with serious bacteria.

Our study aims to identify the bacterial and heavy metal contamination in common herbal medicine products in the central region of Saudi Arabia.

## Methods

### Study design

This study was performed as experimental research to identify the bacterial and heavy metal contamination in complementary herbal medicine products sold in local stores at Qassim region, a central region in Saudi Arabia.

### Study sample

For resources and time reasons, we followed a convenience sampling method and included all local stores of herbal complementary medicine in Qassim region. There are no statistics about number of products available in the region to calculate the needed sample size, so we decided to collect all readily available complementary herbal medicine products which include creams, liquids, and powders. All collected samples were analyzed in the laboratories of the College of Applied Medical Sciences, Qassim University.

### Sample processing and isolation

- Isolation and Identification of Contaminating Bacteria was carried out as defined by Irfan et al. [14]:

The process started by weighing one gram (1 g) of the sample into a sterile Muller Hinton broth and mixed by vortex for 20s, before being incubated at 37 °C for 4 h to enhance microorganism growth. All of the samples were cultured onto Blood agar and MacConkey, streak plate method, and incubated at 37˚C for 24 h. Blood agar was used to grow a wide range of microorganisms and to detect hemolytic bacteria, while MacConkey agar was chosen to isolate gram-negative enteric bacteria and to differentiate bacteria based on their lactose metabolism. Initial identification was performed through colony morphology, gram-stain and biochemical tests.

- Confirmatory identification and determination of antimicrobial susceptibility pattern:

All bacterial isolates were further analyzed using MicroScan WalkAway96 pulse for identification and the antimicrobial susceptibility pattern was determined. The MicroScan WalkAway96 pulse is an automated system which incubates microtiter identification and antimicrobial susceptibility testing panels, interprets biochemical results using a photometric or fluorogenic reader and generates computerized reports. Any bacteria with resistance to more than one antibiotic from different classes was considered multi-drug resistant.

### Evaluation of heavy metal contamination

Elemental analysis was carried out as explained by Zagui et al. [15]. ThermoFisher scientific ARL QUANT’X XRF spectrometer instrument model: AA83811 was used to detect the heavy metals in the selected samples. Thirty-two samples were tested for heavy metal contamination and seven heavy metals were analyzed (Chromium “Cr”, Copper “Cu”, Iron “Fe”, Lead “Pb”, Manganese “Mn”, Arsenic “As” and Zinc “Zn”). The selections of heavy metals was based on their frequent presence in herbal medicine and their toxicity to human health [2, 7].

One gram of each sample was placed in special cups inside the spectrometer chamber facing down toward the X-ray tube. X-rays from the X-ray tube penetrated the layers of the disk and excited the elements in the sample by ejecting inner orbital electrons from the atom by a process known as the photoelectric effect. As the excited atoms return to their ground state, they emit fluorescent X-ray photons with energies that are characteristic of each element in the sample. These X-ray photons are measured by a detector as a mass percentage (m/m%).

### Data analysis plan

All of the collected data were updated and analyzed using to obtain the statistics, frequency, and percentages. Descriptive statistics were done using Microsoft Excel and the results were interpreted in tables using Microsoft Word, and charts using Microsoft Excel.

## Results

**Fig. 1:**
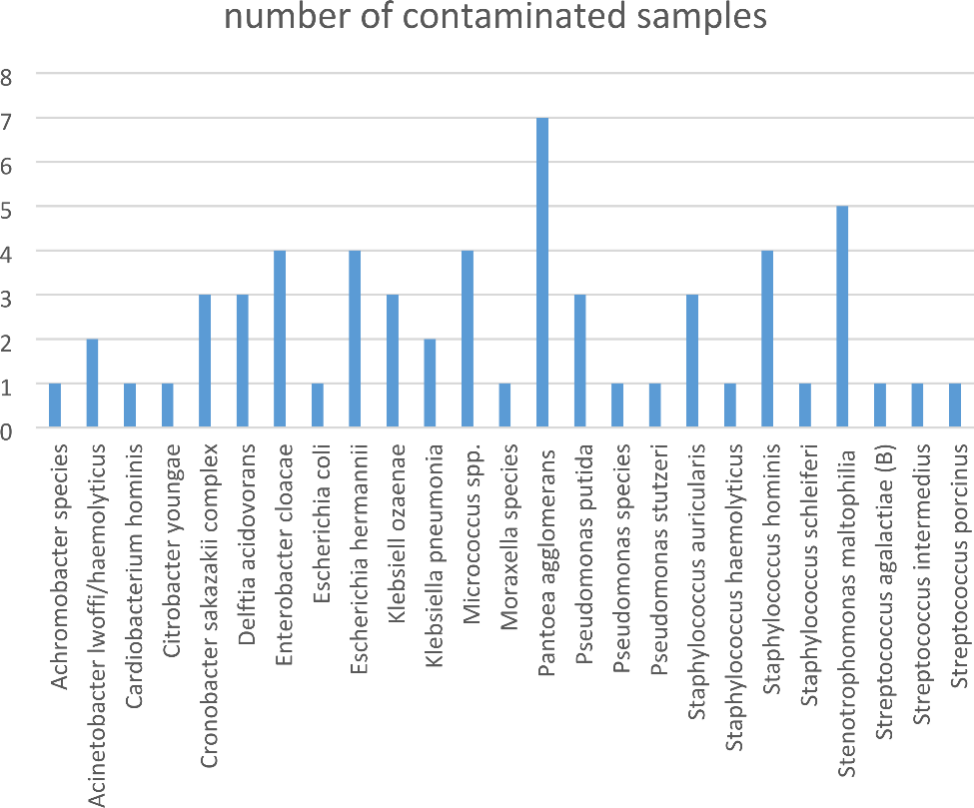
Frequency rate of bacterial isolates from herbal medicine products. Pantoea agglomerans was the most isolated bacteria (7 samples)

A total of 47 samples were collected, including 18 creams, 15 liquids/lotions, and 14 powder products. Most of the products were meant to be applied topically on the skin. Forty four samples were found to be contaminated; only 3 samples did not grow any bacteria in the cultures (Table 1). Overall, 59 bacterial species were successfully isolated and identified (Fig. 1). Nine samples had staphylococcal bacteria (4 *S. hominis*, 3 *S. auricularis*, 1 *S. haemolyticus* and 1 *S. schleiferi*), and 7 had *Pantoea agglomerans*.

**Table 1:** Types of organism isolated from the tested samples of herbal medicine products

Sample number	Isolated bacteria
1	Pantoea agglomerans
2	Pseudomonas species
3	Cronobacter sakazakii complex & Klebsiell ozaenae
4	Pantoea agglomerans & Pseudomonas stutzeri
5	Pseudomonas putida
6	Pantoea agglomerans & Enterobacter cloacae
7	Enterobacter cloacae
8	Pseudomonas putida & Stenotrophomonas maltophilia
9	Pantoea agglomerans & Stenotrophomonas maltophilia
10	Klebsiella pneumonia
11	Acinetobacter lwoffi/haemolyticus & Klebsiella pneumonia
12	Enterobacter cloacae
13	Pantoea agglomerans & Enterobacter cloacae
14	Delftia acidovorans & Escherichia hermannii
15	Klebsiell ozaenae
16	Pantoea agglomerans
17	Cronobacter sakazakii complex
18	Streptococcus agalactiae (B)
19	Pantoea agglomerans & Klebsiell ozaenae
20	Moraxella species & Delftia acidovorans & Stenotrophomonas maltophilia
21	Cronobacter sakazakii complex & Delftia acidovorans
22	Escherichia hermannii & Streptococcus intermedius
23	Citrobacter youngae
24	Acinetobacter lwoffi/haemolyticus
25	Streptococcus porcinus
26	Stenotrophomonas maltophilia
27	Escherichia coli & Staphylococcus schleiferi
28	Achromobacter species
29	Pseudomonas putida
30	Escherichia hermannii
31	Stenotrophomonas maltophilia
32	Cardiobacterium hominis
33	Staphylococcus hominis
34	Escherichia hermannii
35	No growth
36	Micrococcus spp.
37	Staphylococcus hominis
38	Staphylococcus auricularis
39	Staphylococcus auricularis
40	Micrococcus spp.
41	Staphylococcus hominis
42	Staphylococcus haemolyticus + micrococcus spp.
43	Staphylococcus auricularis
44	Micrococcus spp.
45	No growth
46	Staphylococcus hominis
47	No growth

Antibiotic susceptibility testing was then done using the Microscan Walkaway system. This test included 23 different antibiotics. All isolated Staphylococcus spp. were found to be multi-drug resistant (MDR). Staphylococcus hominis was resistant to 10 different antibiotics including ampicillin, erythromycin, and clindamycin. All isolated gram-negative bacteria (42) were found to be multi-drug resistant except three samples, *Citrobacter youngae*, *Cronobacter sakazakii* complex, and *Klebsiella ozaenae*, which were resistant to only one antibiotic. Moreover, *Enterobacter cloacae*, *Pantoea aggiomerans*, and *Stenotrophomonas maltophillia* were found to be resistant to more than 15 different antibiotics including ceftolozane-tazobactam, piperacillin, trimethoprim-sulfamethoxazole and nitrofurantoin.

Thirty-two samples were tested for heavy metal contamination and seven heavy metals were analyzed (Chromium “Cr”, Copper “Cu”, Iron “Fe”, Lead “Pb”, Manganese “Mn”, Arsenic “As” and Zinc “Zn”). All samples had high amounts of heavy metals (Table 2). Iron, manganese and zinc were detected in almost all of the tested samples. The least detected metal was chromium (2 samples) then Arsenic (3 samples).

**Table 2:** Heavy metals concentrations in the tested samples of herbal medicine products

No. of sample	Element ratio in the samples (ppm)						
FDA limit	Chromium 50	Copper	Iron	Lead 20	Manganese	Arsenic 3	Zinc
1					184		209
2			450				
3			876				
4			464				488
5					208		
6			1970				
7			4200				
8			4280				
9	638	63,000	10,200	2930		86,000	2030
10		7270	7090			679	
11			18,600		232		
12	268		10,600		273		206
13			5150		1340		
14			4650		722		275
15			6890		346		307
16			28,900		1400		424
17			1530				662
18			18,300		587		247
19			5630		186		
20			942				
21			12,900		294		394
22			11,100		310		
23		205	5190		256		471
24		1350	18,000		324		
25		232	5020		690		857
26		2850	1790		500		210
27			6670	830	4050	36	627
28			9800		1240		205
29			10,200		1200		365
30			6670		3330		660
31		216			3090		443
32			65,100		742		343

## Discussion

Herbal medicine is the most common form of complementary and alternative medicine. It is commonly used globally with an increasing industrial interest in the recent years. Over 60 billion USD is yearly spent on herbal medicine worldwide [2, 16]. The use of herbal medicine might be of medical or psychological benefit for consumers. However, if strict guidelines and scientific studies are missing, health risks are expected. These concerns are related to microbial and heavy metal contamination. The data from the present study suggest that herbal medicine products are potential sources of microbial contamination, including highly resistant bacteria and heavy metal contamination. The analyzed products included topical creams and powders.

To the best of our knowledge, this is the first time that *Delftia acidovorans*, *Streptococcus porcinus* and *Staphylococcus schleiferi* have been isolated from herbal medicine products. This is alarming for public health as the increased human exposure to new bacteria may contribute to the emergence of new bacterial infections. This can be seen more in immunocompromised patients but also in healthy people [17]. In our study, most of the isolated bacterial species were gram negative bacteria, such as Klebsiella spp., Pseudomonas spp. and Enterobacter spp., in addition to different species of Staphylococci. Comparable results were found in a recent study from Malawi [12]. It has been found that microbial load in herbal medicine products is influenced by the climate, the antimicrobial properties of the plant, the plant surface and the distance of the plant from the soil [13]. Additional post-harvesting, transportation, storage and mixing factors and hygiene should also play a role.

The most commonly isolated species in our study, *Pantoea agglomerans*, is a gram-negative bacillus that is usually found in fecal material, plants and soil. Walusansa et al. found that *E. coli* was the most commonly isolated genus, followed by both Salmonella spp. and Shigella spp., which might indicate fecal contamination [18]. In our study, several species of potentially fecal origin were isolated including *E. coli*, *Pantoea agglomerans*, *Enterobacter cloacae* and *Escherichia hermannii*. Due to the pathogens it carries, fecal contamination is source of several infectious conditions which could sometimes be fatal. This includes gastrointestinal infections, hepatitis, respiratory infections, and skin rashes [19].

The allowed limits in cosmetic products are not defined for most heavy metals; their concentrations are usually measured in parts per million (ppm). The U.S. FDA has defined maximum limits for lead (20 ppm) and arsenic (3 ppm) in cosmetic products, while chromium is allowed in cosmetic colorants in concentrations not exceeding 50 ppm [20]. Heavy metals were detected at very high concentrations in all of the tested samples. Chromium, lead and arsenic were found at levels higher than those defined by the U.S. FDA in 2, 2 and 3 samples, respectively. Iron, zinc and manganese were the predominant metals, but arsenic, lead, chromium and copper were also detected in some samples. The source of these metals could be natural, agricultural, or industrial. Although the concentrations found were surprisingly high, it has been shown that heavy metals are found in herbal products in concentrations much higher than those in synthetic products [21]. Asian herbal medicine products have also shown to contain heavy metals and drugs [22]. When ingested/absorbed, even at low levels, they can be of harm [7]. The long-term accumulation of these metals may lead to cancers, intellectual abnormalities, nephrotoxicity, neurotoxicity, hepatotoxicity, cardiovascular, and skin toxicities [23]. In a recent study, even conventional cosmetic creams may have the same risks [14].

Although the Saudi national center for CAM has published strict regulations for its practice [24], these products were easily reachable through herbal stores or non-licensed traditional healers. The results of the current study show that surveillance might be insufficient. One of the reasons is that these herbal shops are licensed by the ministry of commerce without direct surveillance by the ministry of health or the Saudi FDA. Another more problematic reason is that these products are sometimes sold non officially and distributed in the public, away from the regulatory and surveillance bodies.

A main limitation of the study is that we collected samples from all Qassim region’s local stores. This convenience sampling method might not be well representative of the whole country. We did not have any statistics about number or types of herbal medicine in Saudi Arabia or Qassim region. Consequently, the sample size was not statistically calculated. Another limitation is that we did not replicate samples to ensure consistency of the results. Finally, herbal stores did not declare the ingredients of these mixtures, so, making a link between specific ingredients and the bacterial/metallic load was not possible. We encourage future studies to try to assess these links to have a clearer idea.

## Conclusion

Our study shows the serious level of microbial and metallic contamination of herbal medicine products. What is more concerning is that the isolated bacteria were multi-drug resistant. This might be harmful to the public as it may spread rare and multi-drug bacteria among the local population. Metallic contamination may also lead to cancers and vital organs’ chronic toxicities leading to major health burden. We recommend that all countries should apply strict regulations and continuous surveillance on CAM to prevent malpractice. Joint committees between the ministry of health, the FDA, and the ministry of commerce might help monitoring this practice. Although they could be medically helpful, studies on the efficacy and safety of herbal products are scarce. We recommend financially supporting and encouraging studies on the efficacy and safety of the different CAM practices.

## Data Availability

All data generated or analysed during this study are included in this published article.
